# Preclinical characterization of CPL304110 as a potent and selective inhibitor of fibroblast growth factor receptors 1, 2, and 3 for gastric, bladder, and squamous cell lung cancer

**DOI:** 10.3389/fonc.2023.1293728

**Published:** 2024-01-12

**Authors:** Delfina Popiel, Aleksandra Stańczak, Monika Skupińska, Agata Mikołajczyk, Paulina Stańczak, Filip Mituła, Joanna Hucz-Kalitowska, Kinga Jastrzębska, Damian Smuga, Jakub Dominowski, Monika Delis, Krzysztof Mulewski, Wojciech Pietruś, Daria Zdżalik-Bielecka, Karolina Dzwonek, Monika Lamparska-Przybysz, Abdellah Yamani, Patrycja Olejkowska, Natalia Piórkowska, Krzysztof Dubiel, Maciej Wieczorek, Jerzy Pieczykolan

**Affiliations:** ^1^ Preclinical Development Department, Celon Pharma S.A., Kazuń Nowy, Poland; ^2^ Clinical Development Department, Celon Pharma S.A., Kazuń Nowy, Poland; ^3^ Medicinal Chemistry Department, Celon Pharma S.A., Kazuń Nowy, Poland

**Keywords:** FGFR, FGFR inhibitor, FGFR aberrations, solid tumor, targeted therapy, CPL304110

## Abstract

Fibroblast Growth Factor Receptors (FGFRs) are a family of receptor tyrosine kinases expressed on a plethora of cell membranes. They play crucial roles in both embryonic development and adult tissue functions. There is an increasing amount of evidence that FGFR-mediated oncogenesis is mainly related to gene amplification, activating mutations, or translocation in tumors of various histological types. Dysregulation of FGFRs has been implicated in a wide variety of neoplasms, such as bladder, gastric, and lung cancers. Given their functional significance, FGFRs emerge as promising targets for cancer therapy. Here, we introduce CPL304100, an innovative and highly potent FGFR1–3 kinase inhibitor demonstrating excellent *in vitro* biological activity. Comprehensive analyses encompassed kinase assays, cell line evaluations, PK/PD studies surface plasmon resonance studies, molecular docking, and *in vivo* testing in mouse xenografts. CPL304110 exhibited a distinctive binding profile to FGFR1/2/3 kinase domains, accompanied by a good safety profile and favorable ADMET parameters. Selective inhibition of tumor cell lines featuring active FGFR signaling was observed, distinguishing it from cell lines lacking FGFR aberrations (FGFR1, 2, and 3). CPL304110 demonstrated efficacy in both FGFR-dependent cell lines and patient-derived tumor xenograft (PDTX) *in vivo* models. Comparative analyses with FDA-approved FGFR inhibitors, erdafitinib and pemigatinib, revealed certain advantages of CPL304110 in both *in vitro* and *in vivo* assessments. Encouraging preclinical results led the way for the initiation of a Phase I clinical trial (01FGFR2018; NCT04149691) to further evaluate CPL304110 as a novel anticancer therapy.

## Introduction

Fibroblast growth factors (FGFs) are a family of growth factors that play an important role in tissue repair, regeneration, and differentiation. There are four membrane FGF receptors (FGFRs), namely, FGFR1 through FGFR4, and FGFR4 is the least well-understood among them and has a different kinase domain structure. These receptors are highly conserved transmembrane proteins containing intracellular receptor tyrosine kinase domains. Interaction of various FGF ligands with the receptors activates FGFR-dependent pathways (1–4), which determines further intracellular signal transduction via a plethora of phosphorylation events (5). The FGF/FGFR interactions and subsequent activation of signaling pathways regulate embryogenesis, adult angiogenesis, and wound healing and have a great impact on local tissue homeostasis (6). Since the FGF/FGFR signaling is essential for various physiological processes, the deregulation of FGFR family signaling has a strong effect on tumorigenesis and cancer progression in various human cancers (7). There are several oncogenesis-related FGFR aberrations, including point mutation, copy number variation, gene fusion, and one of the most frequent genetic changes—FGFR amplification (8). For example, *FGFR1* is amplified in lung and breast cancers and rarely in pancreatic and squamous cell lung cancers, whereas *FGFR2* amplification mainly occurs in gastric and breast cancers. Bladder cancer is characterized by genetic aberrations in *FGFR3* (9). Various somatic mutations of FGFR family members also lead to carcinogenesis, such as mutations in *FGFR1* sequence in lung tumors and gliomas, FGFR2 in carcinomas, and FGFR3 in bladder carcinomas and multiple myelomas (10). Overall, a recent study of 4,853 solid tumors showed changes in the genes encoding FGFRs in 7.1% of human cancers, including urinary tract epithelium (32%), breast (18%), endometrium (13%), lung (squamous cell) (13%), and ovarian (9%) cancers (11).

The high frequency of FGFR alterations among many cancer types makes FGFR kinase inhibition a promising therapeutic strategy in a variety of cancers (12). Also, clinical data of the anti-FGFR drugs that are under development have confirmed the results of preclinical studies, suggesting that FGFR plays an important role in some types of cancer and is a well-validated therapeutic target. As an important target, FGFRs attract much interest, and several potent tyrosine kinase inhibitors (TKIs) are currently being tested in clinical trials (13). The current landscape of FGFR inhibitors includes small-molecule receptor TKIs (non-selective, selective, and covalent), monoclonal antibodies, FGF ligand traps, and DNA/RNA aptamers (4). Hence, there are two distinct strategies in new TKI development. The first approach—including erdafitinib and pemigatinib (both registered by the Food and Drug Administration (FDA) for use in the therapy of solid tumors)—involves selective inhibitors, which provide the most potent inhibition of FGFRs. The second approach—presented by nintedanib, regorafenib, and ponatinib—is based on multitarget inhibitors, which also inhibit other kinases, such as VEGFRs and PDGFRs. Still, there is a wide array of new therapeutic molecules that can use both strategies, and several FGFR inhibitors showing potential in preclinical studies *in vitro* and *in vivo* on FGFR-amplified tumors are currently being assessed in Phase I and II clinical trials (4). Nevertheless, in the clinical setting, it is important to unravel what class of agents is the most promising (non-selective or selective FGFR inhibitors), which strategy is more viable, and whether combination therapies are needed to obtain meaningful clinical benefits. Moreover, predictive biomarkers are important in targeted therapies, as they may help physicians choose the right drug for the patient to ensure the greatest benefit from the novel therapy. It has become clear that molecular fingerprints are a great example of prognostic biomarkers, and at every stage of therapeutic intervention, physicians have to consider the molecular fingerprints of a particular cancer before deciding on a proper therapeutic strategy. With targeted therapies, up till now, choosing a combination of novel TKIs is considered the best approach to combat acquired resistance in patients (14).

In this study, we presented the results of the preclinical development of CPL304110—a small-molecule FGFR inhibitor—which is currently under clinical development as a potential drug candidate for patients with FGFR gene aberrations suffering from gastric, bladder, or squamous cell lung cancer. CPL304110 [patent number: WO 2014/141015] is a potent and selective inhibitor of FGFR 1, 2, and 3 receptor tyrosine kinases (15). Its structure is based on a 2-(1*H*-pyrazol-5-yl)-1*H*-benzimidazole pharmacophore and 3,5-dimethoxybenzene moiety specific for other FGFR inhibitors that are currently undergoing Phase I clinical trials. The discovery and the optimization process of 304110 were presented by Yamani et al. (16). The completed preclinical evaluation allowed us to qualify the compound as a clinical candidate for the treatment of FGFR-dependent cancers. CPL304110 is currently being investigated in a first-in-human Phase I clinical trial (NCT04149691; 01FGFR2018).

## Materials and methods

### CPL304110 (CPL110) chemical characterization

**Figure 1:**
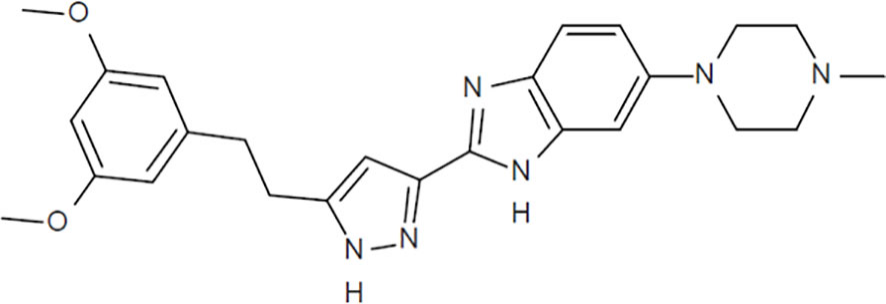
Chemical structure of CPL304110 (MW = 446.54).

2-{5-[2-(3,5-Methoxyphenyl)ethyl]-1*H*-pyrazol-3-yl}-5-(4-methylpiperazine-1-yl)-1*H*-benzo[*d*]imidazole (CPL304110, Celon Pharma; **Figure 1**) was synthesized in accordance with the processes described in the International Patent Application Publication Number WO 2014/141015, 2014, A1 (15). Compound CPL304110 as a free base is soluble under physiological pH (1.2–7.4) in the range of 0.01–40 mg/mL. Distribution coefficients (logD) strongly depend on pH (0.44 for logD 1.2 and 4.22 for logD 7.4). For *in vitro* studies, CPL304110 was prepared as a 10 mM stock solution in dimethyl sulfoxide (DMSO) and diluted in the relevant assay media. For *in vivo* studies, CPL304110 was formulated in a 2% NMP/33% PEG300/65% H_2_O (*v/v*) solution.

### Analysis of metabolic stability

Assessment of metabolic stability was performed as described previously (17) using human (HLM) and mouse (MLM) liver microsomes (Phase I) and mouse hepatocytes (MH) (Thermo Fisher Scientific, Waltham, MA, USA). The experiment was performed in four (microsomes) or three (hepatocytes) biological replicates, each in triplicate. Verapamil and warfarin (Merck, Darmstadt, Germany) were used as high- and low-clearance reference compounds, respectively. The elimination rate constant (*k*) was calculated as a slope module of log-linear regression of the substrate content *vs.* time. Then, intrinsic clearance (*Cl*_int_) was calculated as *Cl*_int_ = *k*/*d*, where *d* is a protein or cell density for microsomal or hepatocyte stability. Compounds’ disappearance from the reactions was analyzed using an ultrahigh-performance liquid chromatography (UHPLC) system (Vanquish Flex, Thermo Fisher Scientific) coupled to a Triple Quad MS detector (QTRAP 5500+, Sciex, Framingham, MA, USA). LC separation was achieved using an ACQUITY UPLC BEH C18 50 × 2.1 mm, 1.7 μm column (Waters, Milford, MA, USA) at 50°C. The mobile phases were (A) 0.1% (*v*/*v*) formic acid in water and (B) 0.1% (*v*/*v*) in acetonitrile. Samples were eluted using a mixed mode elution starting with the isocratic flow at 5% B from 0 to 0.5 min, followed by a linear gradient to 95% B at 2.0 min, held at 95% B until 3.0 min, and then back to the initial 5% B at 3.2 min. The columns were equilibrated for up to 4.5 min. The flow rate was 0.5 mL/min. The injection volume was 2 μL. Detailed mass spectrometry (MS) detection parameters are presented in **Supplementary Table 1**.

### hERG channel binding

The affinity of CPL304110 to the hERG potassium channel was investigated using a hERG Predictor™ fluorescence polarization kit (Invitrogen, Carlsbad, CA, USA; PV5365) in 384-well plate format, as described in the manual provided by the vendor. E-4031, a potent hERG blocker, was used as a reference compound to validate the assay’s performance. Sixteen-point dilution series were prepared at concentrations of 30 μM to 2.1 pM using threefold dilutions. Fluorescence was read using a Victor Nivo spectrofluorometer (PerkinElmer, Waltham, MA, USA), and the polarization was calculated in accordance with the instrument manual, with the G-factor determined experimentally as 0.9048. IC_50_ of hERG binding was calculated using constrained four-parameter non-linear regression (log(inhibitor) *vs.* response) in GraphPad Prism 8 software.

### Inhibitor activity

The inhibitory activity of CPL304110 against tested proteins was evaluated with ADP-Glo assay (Promega, Madison, WI, USA) following the manufacturer’s protocol. CPL304110 was dissolved in 100% DMSO and used as a stock solution, and serial dilutions were prepared using dilution buffer (20 mM Tris pH 7.5, 10 mM MgCl_2_, 0.1 mM Na_3_VO_4_, 0.01% Triton X-100, and 2.5 mM DTT). Kinases FGFR1, FGFR2, and FGFR3 (Carna Biosciences, Kobe, Japan) were diluted in buffer (50 mM Tris-HCl pH 7.5, 150 mM NaCl, 10% glycerol, 0.05% Triton X-100, and 1 mM DTT) to final concentrations of 0.5 ng/μL, 0.4 ng/μL, and 1 ng/μL, respectively. Luminescence intensity was measured using a Victor Light luminometer (PerkinElmer, Inc.). IC_50_ values were determined in GraphPad Prism 8 software by fitting to individual points of the curve by non-linear regression (log(inhibitor) *vs.* normalized response - Variable slope). Each compound was tested in at least six technical replicates in two separate experiments.

### Cell lines

To assess the cytotoxicity of the compounds, various cancerous and control cell lines were used (**Supplementary Table 2**). In all studies, two groups of cell lines were used, one group dependent on FGFR signaling with confirmed genetic FGFR background (NCI-H1581, SNU-16, RT-112/84, KATO III, UM-UC-14, AN3CA, KMS 11, SW-780, NCI-H520, and CAL120) and another group not dependent on FGFR signaling (WSU-NHL, HEL 92.1.7, HepG2, HCT116, HeLa, U937, T24, LoVo, H460, and PC-3). A human umbilical vein endothelial cell (HUVEC) line was also used as a control. Detailed information about the used cell lines is shown in **Supplementary Table 2**. All cell lines were cultured following manufacturers’ instructions and were confirmed negative for mycoplasma (Venor®GeM qEP Mycoplasma Detection Kit for qPCR, Minerva Biolabs, Berlin, Germany). Selected cell lines with proven tumorigenicity were also used for *in vivo* studies. To select an appropriate statistical test, normal distribution analysis was performed in the groups using the Shapiro–Wilk test. Due to the lack of normal distribution, a non-parametric Mann–Whitney U test was performed to compare IC_50_ values between the two groups: cell lines with FGFR aberrations and cell lines with FGFR wild-type group.

### Analysis of cell proliferation

The antiproliferative activity of CPL304110 was determined in a panel of human tumor cell lines of different origins and FGFR signaling dependence status and the control non-neoplastic cell line, HUVEC. Cells were treated for 72 h with a dilution series (e.g., 21.01 > 6 > 1.72 > 0.49 > 0.14 > 0.04 > 0.02 > 0.01 > 0.005 µM) of CPL304110, and cell viability was tested using ATPlite Luminescence Assay (PerkinElmer) following the manufacturer’s instructions. The experiments were carried out in two biological replicates. IC_50_ was determined using GraphPad software.

### Compound interaction with kinases

The KINOMEscan™ screening platform (DiscoverX) was used to measure interactions between our compound CPL304110 and a panel of human kinases and disease-relevant mutant variants. The analyses were performed using DiscoverX in line with the service provider’s protocol. In this binding assay, the tested compound in solution was incubated with DNA-tagged kinase and kinase active site-directed ligand bound to the bead. The compound competed for binding to kinase with the immobilized ligand. After incubation, the beads were removed from the solution, and kinase bound to the ligand was quantified by qPCR. The strength of compound binding was determined based on its ability to block kinase–ligand binding (18). Eleven-point twofold serial dilutions of CPL304110 were used to determine its percentage of inhibition of different kinases. Analyses were performed in two independent experiments, for the compound concentration of 100 nM on the 100-kinase panel and the compound concentration of 1,000 nM on the 468-kinase panel. The measure of compound affinity to each kinase in the panel was the percentage of kinase activity inhibition by the tested ligand in comparison to the control in both experiments.

### Surface plasmon resonance measurements

The surface plasmon resonance experiments were performed using a Biacore S200 system (Cytiva, Marlborough, MA, USA) equipped with a CM5 sensor chip. The ligands FGFR1, FGFR2, and FGFR3 (Carna Biosciences) at a concentration of 20 μg/mL in 10 mM sodium acetate, pH 4.5, were immobilized using amine-coupling chemistry at densities of 17,050 RU (300 s), 17,150 RU (400 s), and 12,950 (550 s) on flow cells 2, 3, 4, respectively, with flow cell 1 left blank for reference. Kinetics analysis was performed in running buffer (10 mM HEPES, 150 mM NaCl, and 0.01% Tween20, pH 7.4), and data were collected at a rate of 1 Hz. Triplicate injections of CPL304110 at concentrations of 50 nM, 16.7 nM, 5.6 nM, 1.85 nM, 0.61 nM, and 0.2 nM and a triple buffer blank were injected in single-cycle mode over all four surfaces simultaneously at a flow rate of 30 μL/min and a temperature of 25°C. The complex was allowed to associate for 60 s and dissociate for 300 s, with no regeneration steps. The data were fit to a simple 1:1 interaction model using the global data analysis option available within Biacore S200 Evaluation Software 1.1.1 (Cytiva).

### Docking studies

The crystal structures of FGFR proteins FGFR1 (PDB ID: 4RWJ) (19), FGFR2 (PDB ID: 7OZY) (20), and FGFR3 (PDB ID: 7DHL) (21) were retrieved from the Protein Data Bank (22, 23). The 3D ligand structures were prepared using Instant JChem 21.13.0 software, with appropriate protonation states based on calculated pK_a_ values. The PropKa program was used to identify the protonation state of the amino acids (24). The grid was centered on crystalized inhibitors in crystal structures. The visualizations of obtained binding modes were prepared using PyMOL software.

### Binding constant (Kd) determination

Analyses were performed using DiscoverX. Binding constants (Kd) for the CPL304110 compound were determined for relevant kinases selected for the KINOMEscan profiling. In 100% DMSO, 100× compound dilutions were prepared and subsequently diluted to 1× in the assay (with a final DMSO concentration of 1%). Eleven-point threefold serial dilutions were used. Most Kd values were determined using a compound top concentration of 30 µM. If the initial determined Kd was lower than the lowest concentration tested (0.5 nM), the measurement was repeated with a serial dilution at a lower top concentration. A Kd value reported as 40 µM indicates that the Kd was determined to be above 30 µM. The Kd values were calculated using a standard-dose response curve using the Hill equation: Response = Background + Signal − Background/1 + (KdHill Slope/DoseHill Slope) (25). The Hill Slope was set to −1. Curves were fitted using a non-linear least-square fit with the Levenberg–Marquardt algorithm.

### Western blotting analysis

The effects of CPL304110 on intracellular signaling in FGFR-dependent cell lines were assessed using Western blotting. The levels of FGFR receptors and ERK1/2 phosphorylation were determined. β-Tubulin and GAPDH were used as the internal procedure controls. Cellular or tissue lysates were prepared in a lysis buffer, cleared by centrifugation, and normalized for total protein by bicinchoninic acid (BCA) assay (Pierce) in accordance with the manufacturer’s manual. Protein lysates were analyzed using sodium dodecyl sulfate–polyacrylamide gel electrophoresis (SDS-PAGE) gels in a Mini-Protean III System (Bio-Rad Laboratories, Hercules, CA, USA). Western blotting analysis was performed using the following primary antibodies: anti-FGFR1 (D8E4, #9740), FGFR2 (D4H9; #11835), FGFR3 (C51F2, #4574S), anti-ERK (p44/42 MAPK (Erk1/2), #9102), and anti-pERK (p44/42 MAPK, #4370). The secondary antibody was anti-rabbit IgG horseradish peroxidase-conjugated (#7074 Cell Signaling Technology, Danvers, MA, USA). For analysis of β-tubulin, mouse anti-β-tubulin (Millipore, Billerica, MA, USA; #05-661) and a secondary antibody (Sigma, Darmstadt, Germany; A9044) were used. Target proteins were visualized using a LumiLight substrate (Roche, Basel, Switzerland) and then exposed to Light Film BioMax (Kodak, Rochester, NY, USA) in line with the manufacturer’s instructions.

### BioMAP profiling

BioMAP profiling was conducted using DiscoverX following the service provider’s protocol. In this experiment, the compound CPL304110 was characterized in four concentrations (10,000 nM, 3,300 nM, 1,100 nM, and 370 nM) on the panel of 12 human primary cell-based systems designed to model different aspects of the human body in an *in vitro* format. Data were analyzed from cells and stimulators from the systems shown in **Supplementary Table 3**. Significant biomarker readouts were annotated, and these key activities were classified and listed into biologically relevant categories. Profile plots could identify dose-dependent, cytotoxic, antiproliferative, and potential off-target secondary effects. The profile plots were followed by an overlay of one concentration of the test agent with one concentration of a selected Reference Benchmark. The expression of biomarkers specific to each system was measured using reference techniques. Results were presented as the log-transformed ratio of the biomarker readings for the drug-treated sample to the vehicle control. Biomarker activities were considered altered when there were two or more sequential concentration shifts in the same direction relative to the vehicle control, they were outside of the significance envelope, or they had at least one concentration with an effect size >20%.

### SAFETYscan47

The SAFETYscan47 test was performed using DiscoverX following the service provider’s protocol. Genetically modified reporter cell lines were treated with CPL304110 at 10 µM. G-protein-coupled receptor (GPCR), transporter, ion channel, nuclear receptor, kinase, and other non-kinase enzyme activities were measured in the corresponding cell model (**Supplementary Table 4**). The effect of our compound was measured as a percent of response under control conditions.

### Cell line xenograft models

All animal experiments were performed at the Animal Laboratory of Hirszfeld Institute of Immunology and Experimental Therapy in Wroclaw (Poland), with approval from the Local Ethics Committee for Treatment of Laboratory Animals in Poland and in line with “3R” guidelines. The experiments were performed under the Guidelines for the Care and approved protocols by the Local Ethics Committee for Treatment of Laboratory Animals in Wroclaw (Poland), according to Resolutions no. 15/2013, 50/2013. Animal welfare is a value of the Union that is enshrined in Article 13 of the Treaty on the Functioning of the European Union (TFEU). The use of animals in procedures followed the international law: “DIRECTIVE 2010/63/EU OF THE EUROPEAN PARLIAMENT AND OF THE COUNCIL of 22 September 2010 on the protection of animals used for scientific purposes”. All protocols approved by the Local Ethics Committee are reviewed in this regard. Eight-week-old female SCID mice (CB17/Icr-Prkdc^SCID^/IcrlcoCrl) were obtained from Charles River (Wilmington, MA, USA). Tumors were established by subcutaneous injection into the left flank using 0.1 mL tumor cells suspended in phosphate-buffered saline (PBS) (5 × 10^6^ for SNU16, 1 × 10^7^ for H1581, 1 × 10^6^ for RT-112, and 5 × 10^6^ for UM-UC-14) mixed 3:1 with Matrigel (BD Biosciences, San Jose, CA, USA). Mice were randomized into control and treatment groups (n = 6 mice/group) when tumors reached the determined size of approximately 100 mm^3^. For PK/PD studies using the RT-112 tumor model, the animals were randomized into five individual groups. Tumor volume (measured by Vernier caliper), animal body weight, and tumor condition were recorded every 2 days for the overall duration of the study. Tumor volume (TV) was calculated using the formula TV = (L × (W^2^))/2, where L is the largest measurement and W is the smallest measurement. Percent tumor growth inhibition (%TGI) was calculated using the following formula: TGI [%] = [1 − [mean tumor volume of drug-treated group/mean tumor volume of the vehicle-treated control group)] × 100%. Tumor growth inhibition >50% was considered meaningful [Ubezio PB, 2019]. In all the experiments, the animals were dosed orally every 12 h for 14 days (SNU-16 model), 17 days (RT-112 and H1581 models), or 21 days (UM-UC-14 model). Tolerability was estimated by monitoring body weight loss, clinical signs, and survival.

### PK/PD

Tumor and blood samples were collected from RT-112 subcutaneous xenograft tumor-bearing mice at various time points after a single oral dose of either CPL304110 (20 mg/kg, 40 mg/kg) or vehicle. Tumor tissues were collected after the animals’ sacrifice and frozen via liquid nitrogen in two separate pieces. One piece of each tumor sample was homogenized in water, and the other piece was lysed in 1× cell lysis radioimmunoprecipitation assay (RIPA) buffer (Sigma-Aldrich) containing phosphatase inhibitors (PhosSTOP, #04906837001, Roche), protease inhibitors (Halt Protease Inhibitor Cocktail, #78425, Thermo Fisher), and 0.5 M EDTA (#R1021, Thermo Fisher) using a Fast Prep Homogeniser (MP Biomedicals, Santa Ana, CA, USA). Western blotting was conducted as outlined in the Materials and Methods. After the lysis, water-homogenized samples were subjected to deproteinization and used for the determination of the concentration of the compound in both the tumor tissue and the plasma samples. The concentrations were determined using ultrahigh-performance liquid chromatography coupled with mass spectrometry (UHPLC-MS) with electrospray (ESI) ionization and quadrupole and time-of-flight (Q-TOF) analyzers. Western blotting was conducted as outlined in the Materials and Methods.

### Patient-derived tumor xenograft model

Two patient-derived tumor xenograft (PDTX) models with FGFR2 amplification, including HuPrime® GA1224, a gastric adenocarcinoma PDTX model, and HuPrime® LU6429, a non-small cell lung cancer (NSCLC) PDTX model, derived and proprietary to Crown Biosciences, Inc. (San Diego, CA, USA), were chosen. The protocol and any amendments or procedures involving the care and use of animals in this study were reviewed and approved by the Institutional Animal Care and Use Committee (IACUC) of Crown Biosciences before the initiation of the studies. During the study, the care and use of animals were in accordance with the regulations of the Association for Assessment and Accreditation of Laboratory Animal Care (AAALAC). LU6429 and GA1224 tissue portions of primary human tumors were implanted subcutaneously in both flanks of one or two 6–8-week-old female mice (MF-1 nude or BALB/c nude) in accordance with the Crown Biosciences protocols. When tumors reached the exponential growth phase, they were removed from donor mice, divided into small fragments, and serially transplanted to the right flank of new recipient mice. When the mean tumor volume approached over ~100 mm^3^, the mice were randomized into treatment groups (n = 10 mice/group) using a stratified randomization method. There was no significant difference in tumor volume among treatment groups (p > 0.99, one-way ANOVA, groups 1–4 for each PDTX model). The day of randomization was denoted as day 0. The animals were dosed orally with vehicle or CPL304110 twice daily at 12-hour intervals for three consecutive weeks, followed by a 7-day recovery period. Tumor size was measured three times weekly. TV and %TGI were used for the evaluation of antitumor efficacy. For tumor volume reduction was computed using the equation T/T0 (%) = 100 × ΔT/T0, where ΔT is the change in tumor volume in the treatment group (26). Other parameters, such as body weight, physical appearance, behavior, and clinical changes, were also monitored during the studies.

### Statistical data analysis

GraphPad Prism software (version 7) was used for all analyses of *in vitro* and *in vivo* experiments. The statistical significance of differences between the control and treated samples for the PK/PD analyses was determined using one-way ANOVA. The Kaplan–Meier survival curves were generated, and the log-rank test was used to determine survival in the PDTX analysis. The p-values were considered statistically significant (* p < 0.05; ** p < 0.01; *** p < 0.001).

## Results

### CPL304110 is a potent and selective inhibitor of FGFR 1, 2, and 3 *in vitro*

**Figure 2:**
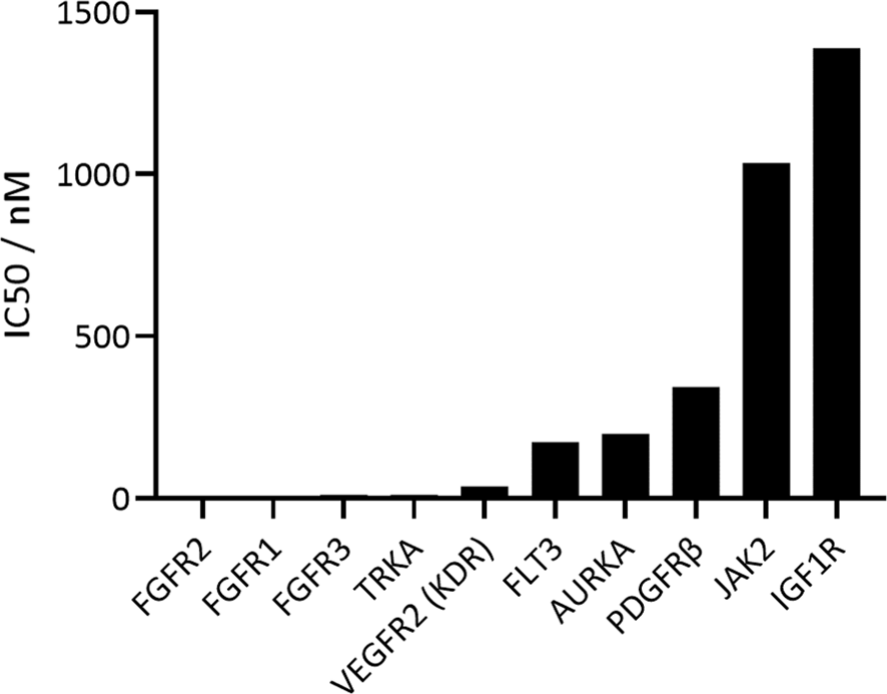
*In vitro* inhibitory profile of CPL304110 with the use of ADP-Glo Kinase Assay.

The activity and selectivity of CPL304110 were first tested on a narrow-selected kinase panel. First, FGFR kinases were tested, and CPL304110 showed the strongest inhibitory activity on FGFR2 (IC_50_ = 1.44 nM), followed by FGFR1 (IC_50_ = 4.08 nM) and FGFR3 (IC_50_ = 10.55 nM). Next, CPL304110 selectivity was analyzed on seven kinases; the results showed that CPL304110 had the highest activity against kinases that share homology with FGFRs (KDR and PDGFRβ) and others that play an important role in tumor development and progression (AURKA, FLT3, IGF1R, JAK2, and TRKA). The highest inhibitory activity was determined for TRKA kinase (IC_50_ = 11 nM) and KDR (VEGFR2) (IC_50_ = 37 nM) (**Figure 2**); however, these IC_50_ values were 7.6- and 25.7-fold higher, respectively, than IC_50_ for FGFR2. The remaining kinases were inhibited by CPL304110 with slightly lower potency; i.e., the IC_50_ values were more than 100-fold higher than IC_50_ for FGFR2. The inhibitory effects on TNIK, GSK3β, CDK2, PIM1, EGFR, and PI3Kα were considered irrelevant because the percentage of inhibition with 1 µM of CPL304110 was less than 5%.

**Figure 3:**
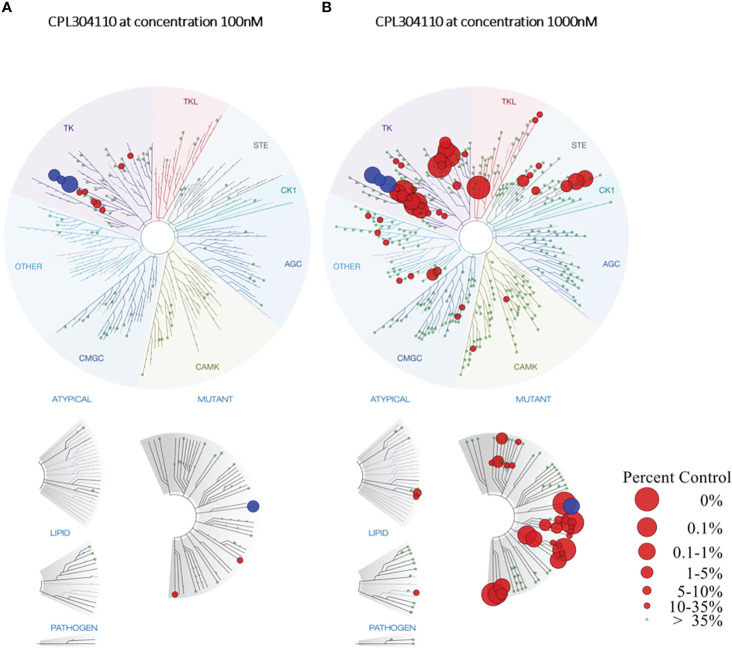
TREE*spot*™ Interaction Maps for CPL304110 at 100 nM **(A)** and 1000 nM **(B)**. Inhibited kinases with <35% of control are highlighted on the graph in red, while wild-type or mutated FGFR1–3 receptors are highlighted in blue. Protein Kinase groups presented: TK – Tyrosine Kinase Group; TKL – Tyrosine Kinase-Like Group; STE – STE Kinase Family Group; CK1 – Cell Kinase 1 Group; AGC – Kinase A, G, and C Group; CAMK - Calcium/Calmodulin-Dependent Kinase Group, CMGC – Group including Cyclin-Dependent Kinases (CDKs), Mitogen-Activated Protein Kinases (MAPK), Glycogen Synthase Kinases (GSK) and Cyclin-Dependent Kinase-Like Kinases.

Finally, a broad *in vitro* screening has been conducted to understand the kinase selectivity profile of CPL304110. The KINOMEscan™ screening platform covering selected 100 human kinases and cancer-relevant mutants was used to determine the selectivity of CPL304110 at 100 nM (data are shown as TREEspot ™, **Figure 3A**). The compound was screened at one concentration, and the results for binding interactions were reported as “% Ctrl”, where lower numbers indicate stronger hits in the matrix. This method does not require ATP and thereby reports true thermodynamic interaction affinities, as opposed to IC_50_ values, which can depend on ATP production. In this study, three of the 100 tested kinases demonstrated ≥90% inhibition of their activity after treatment with 100 nM CPL304110 (i.e., FGFR1, 0.95% of Ctrl; FGFR3, 3.9%; CSF1R, 7.4%). This analysis allowed the identification of the pool of kinases inhibited by the tested compound, of which many are involved in cancer development and progression. Some of the analyzed kinases have been described as tumorigenic factors (e.g., FLT1, KIT, and RET), some are tyrosine kinases responsible for neuroplasticity regulation (TRKA), and one of the kinases has no oncogenic properties (CSF1R). Overall, the targeting profile of CPL304110 strongly supports its potential anticancer activity.

**Figure 4:**
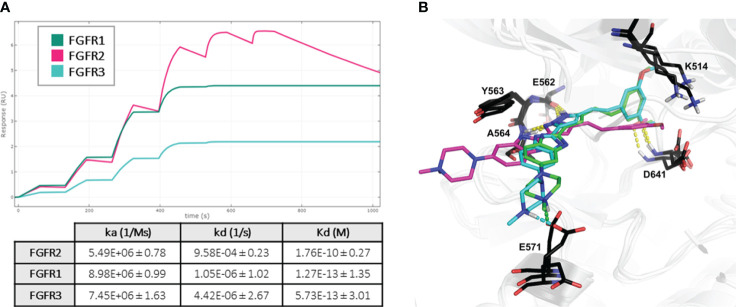
Mechanism of CPL304110–FGFR complex formation. **(A)** CPL304110 shows much lower dissociation rates when interacting with FGFR1 and FGFR3 than with FGFR2. Fit representation was prepared using Biacore™ Simul8 software (Cytiva), based on three replicates. Fitted values are presented in the table. **(B)** Superposition of the binding models of CPL304110 compound in FGFR1 (green), FGFR2 (magenta), and FGFR3 (cyan) catalytic pockets. The hydrogen bonds are marked in yellow dashes, and salt bridges are marked in green and cyan (FGFR1 and FGFR3, respectively).

To gain insight into the binding profile of CPL304110 with FGFR1, FGFR2, and FGFR2, the kinetics of complex formation were analyzed using surface plasmon resonance (SPR). This analysis revealed the kinetic selectivity of CPL304110, with subnanomolar Kd values (FGFR1 = 0. 127 pM, FGFR2 = 176 pM, and FGFR3 = 0.57 pM) and different binding profiles of FGFR isomers. CPL304110 presented a high on-rate and fastest dissociation for FGFR2 (ka = 8.98 × 10^6^ [1/Ms]; kd = 1.05 × 10^−6^ [1/s]). For FGFR1 and FGFR3, slower association (8.98 × 10^6^ and 7.45 × 10^6^ [1/Ms], respectively) coupled with extremely long residence time (1.05 × 10^−6^ and 4.42 × 10^−6^ [1/s]) was documented (**Figure 4A**). Of note, such extremely low dissociation times (<10^−5^ M/s) are outside the limits that can be measured by the instrument, so these cannot be precisely determined and could affect overall Kd values for FGR1 and FGFR3.

To further investigate this behavior, the molecular mechanism of CPL304110 action was evaluated using molecular docking studies to FGFR1 (PDB ID: 4RWJ), FGFR2 (PDB ID: 7OZY), and FGFR3 (PDB ID: 7DHL). The obtained binding mode of CPL304110 in FGFR1 was coherent with our previously reported results (16). In all FGFR isomers, amino acids of the hinge region (E562, Y563, and A564) were involved in hydrogen bonds (HB) with pyrazole fragment, and gatekeeper residue (D641) formed an HB with the methoxy group (**Figure 4B**). However, in FGFR1 and FGFR3, the dimethoxyphenyl fragment was engaged in cation π interaction with K514, whereas in FGFR2 catalytic pocket, this fragment was flipped, and the methoxy group formed a charge-assisted HB (**Figure 4B**). Finally, the *N*-methylpiperazine moiety of CPL304110 was extended to the solvent region (16), which may be crucial for the different binding profiles of CPL304110 with isomers of FGFR. In FGFR1 and FGFR3, the positively charged nitrogen atom of piperazine formed a salt bridge (SB) with E571, whereas this fragment was exposed to the solvent in FGFR2 binding pocket. SB corresponds to the double charge-assisted hydrogen bond [(±)CAHB] and is defined as a bond formed by an acid and a base with close donor–acceptor pKa matching, which makes it the strongest among all known non-covalent molecular interactions (27).

**Figure 5:**
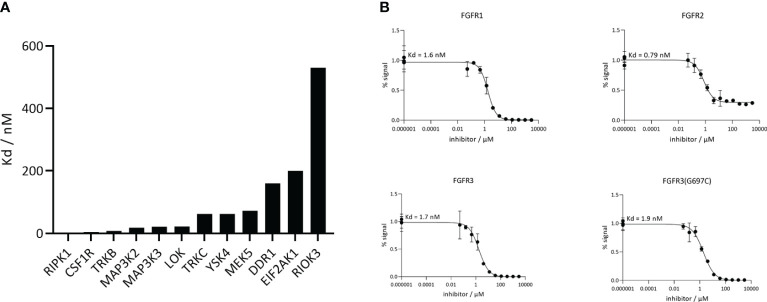
CPL304110 is much more selective toward FGFRs than other kinases. **(A)** Kd values of CPL304110 for the selected additional kinases acquired using DiscoverX. **(B)** Analysis of CPL304110 dissociation rate constant (Kd) for FGFR1, 2, and 3. The amount of kinase measured by qPCR (Signal; y-axis) was plotted against the corresponding compound concentration in nM in log10 scale (x-axis).

The next analysis of CPL304110 included dissociation rate constant (Kd) for FGFR1, FGFR2, FGFR3, FGFR3(G697C) and 12 the most inhibited kinases (CSF1R, DDR1, EIF2AK1, LOK, MAP3K2, MAP3K3, MEK5, RIOK3, RIPK1, TRKB, TRKC, YSK4) selected based on KINOMEscan as an alternative method to confirm the activity and selectivity of our compound. These data showed that the tested compound was more selective for FGFR kinases than for other kinases (**Figure 5A**), as Kd values for FGFRs were much lower, ranging from 0.79 to 1.6 nM (**Figure 5B**), and correlated with the previously determined ones for FGFR 1, 2, and 3 (wild type and mutant).

As the final step, a broad *in vitro* screening against a panel of 468 kinases was conducted for CPL304110. In this study, a 1,000-nM concentration of the compound was used to determine potential off-targets. For FGFR1, FGFR2, FGFR3, and 38 other kinases, the calculated inhibition value was ≥90% (**Figure 3B**). This panel also included kinases structurally similar to FGFR, such as KDR (VEGFR2) and PDGFR. CPL304110 compound had the strongest inhibitory activity (0%–0.1% of control) toward CSF1R, FLT3 (D835V), KIT (A829P), RET, RIPK1, TRKA, TYK2, KIT (V559D), and RET (M918T). Moreover, CPL304110 inhibited the FGFR3 variant with activating G697C mutation that was observed in 62% (44/71) of the examined oral squamous cell carcinoma (OSCC). It is possible that the activating potential of this mutation could be context-dependent and may involve interaction with other genes (7, 9).

### CPL304110 inhibits the proliferation of FGFR-dependent cancer cell lines

**Figure 6:**
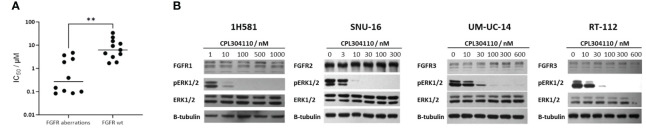
Impact of CPL304110 on different cell lines. **(A)** Changes in cell proliferation after treatment with CPL304110 were determined by ATPlite Luminescence Assay. IC_50_ values of CPL304110 for human cell line proliferation were calculated after treatment with the indicated concentrations of CPL304110 for 72 h. Shapiro–Wilk test was used to test normality, and both groups did not pass normality (p = 0.0076 for the FGFR aberration group and p = 0.0218 for the FGFR wt group), and a non-parametric Mann–Whitney U test was performed to compare IC_50_ values between the two groups. p-Value obtained in the analysis was 0.0015 (**). **(B)** Phosphorylation of ERK 1/2 (p-ERK 1/2) and FGFR2 or FGFR3 levels in selected FGFR-dependent cancer cell lines upon CPL304110. SNU-16, RT-112, UM-UC-14, and H1581 were incubated with the indicated concentrations of CPL304110 for 24 h. β-Tubulin served as a loading control.

Multiple studies have already shown that activation of the FGFR signaling cascade is involved in the proliferation of tumor cells (7–9, 11). Thus, the antiproliferative activity of CPL304110 was determined in a panel of human tumor cell lines of different origins that are dependent on FGFR signaling. To determine the potential *in vitro* safety margin, the effect of CPL304110 on cell proliferation was also studied on a normal, non-neoplastic cell line, HUVECs. The obtained IC_50_ values are summarized in **Figure 6A**. The strongest inhibition of the proliferation potential was found for lung, gastric, bladder, and endometrial cancer cell lines (ranging from 0.084 to 0.393 µM). These cell lines are dependent on the FGFR pathway, with confirmed FGFR aberrations (amplification, mutations, and gene fusions). For the other lines with aberrant FGFR signaling, IC_50_ values were between 1.867 µM and 4.71 µM. The IC_50_ generated for the HUVEC line was very high—over 21 µM. Altogether, these analyses showed that CPL304110 potently inhibits the proliferation of FGFR-dependent cancer cell lines in comparison to cancer cell lines without FGFR aberrations.

Next, the effect of CPL304110 on the intracellular FGFR-dependent signaling network was evaluated in FGFR-susceptible cell lines using Western blotting. The results for selected FGFR-aberrant cancer cell lines (SNU-16, RT-112, UM-UC14, and H1581) are shown in **Figure 6B**. The inhibition of protein phosphorylation was observed for CPL304110 at concentrations as low as 30 nM. Similar results were also confirmed for other cell lines dependent on the FGFR signaling (i.e., NCI-H1581 and AN3CA; data not shown). In all tested cell lines, a 100-nM concentration of CPL304110 demonstrated complete inhibition of pERK1/2 (**Figure 6B**). These data indicated that CPL304110 potently blocks FGFR signaling at the cellular level.

### CPL304110 possesses good drug-like properties

As a pyrazole-benzimidazole derivative, CPL304110 possesses good lipophilicity/hydrophobicity properties in the range between 0.44 and 4.22 logD, at pH 1.2 and 7.4, respectively (**Supplementary Table 5A**). Molecular properties (rotatable bonds, 7; MW, 446.54 Da; HBA, 8; HBD, 2; clogP, 3.34) of CPL304110 meet Lipinski Ro5 criteria and suggest that CPL304110 is a promising drug candidate. The presence of a methylpiperazine residue as a solvent group and the presence of acceptor–donor functions in CPL304110 are responsible for its good solubility in the range of 0.01–40 mg/mL under the physiological pH (1.2–7.4) (**Supplementary Table 5A**). For the determination of passive diffusion as a factor determining the transport through the gastrointestinal tract, penetration of the blood–brain barrier, and transport across cell membranes, a parallel artificial membrane permeability assay (PAMPA) has been performed. However, CPL304110 showed a low permeability (0.81 × 10^6^ cm/s) in a non-cell parallel artificial membrane permeability assay (**Supplementary Table 5A**).

Metabolic stability is an important part of absorption, distribution, metabolism, and excretion (ADMET) research and allows to exclude compounds with low metabolic stability at an early stage of drug development, thereby reducing the risk of potential clinical trial failure (28, 29). Microsomal stability of the test compound, CPL304110, was assessed in mouse and human liver microsomes to determine Phase I *Cl*_int_ and, subsequently, in mouse hepatocytes prior to the *in vivo* studies. In both experiments, verapamil and warfarin were used as reference compounds with poor and good metabolic stability, respectively. *Cl*_int_ of CPL304110 determined in HLM was nearly fourfold lower than of verapamil (**Supplementary Table 6A**), similar to what was observed in MLM. These results suggest that CPL304110 is metabolized at a moderate rate in microsomes during Phase I reactions. In mouse hepatocytes, CPL304110 presented high clearance (70.6 ± 5.4 µL × min^−1^·10^−6^ cells), close to that observed for verapamil (77.5 ± 4.9 µL·min^−1^·10^−6^ cells). Metabolically stable warfarin presented *Cl*_int_ of 1.7 ± 1.5 µL·min^−1^·10^−6^ cells. Although the test compound presented high clearance in hepatocytes, comparable to the known high-clearance reference compound, the obtained *in vitro* data provided strong evidence of its efficacy, which favored *in vivo* testing. Therefore, despite low metabolic stability *in vitro*, CPL304110 was subjected to PK studies to assess its PK profile in mice prior to efficacy studies using xenografts.

To evaluate the anticipated cardiovascular effects of CPL304110, the affinity of CPL304110 to hERG potassium channels was assessed. The affinity was documented at 10.0 ± 8.9 μM (**Supplementary Table 6B**). The assay performance was validated by determining hERG binding IC_50_ using E-4031 (38 ± 21 nM), which was in line with the value of 39 nM declared by the kit manufacturer. Safety margins for a compound to avoid *torsade de pointes* or QT-interval prolongation have been recently reviewed to be 37- and 50-fold lower concentrations than the compound’s therapeutic free plasma concentration (30), that is, 270 nM or 200 nM for CPL304110. Therefore, the safety margin for QT-interval prolongation (only the most stringent margin was taken into account for the calculation) of the compound’s total concentration in human plasma was 46.5 μM (or 20.8 μg/mL) (**Supplementary Table 6B**). In PK studies in mice, the PK profile of CPL304110 administered at an effective dose (40 mg/kg, *p.o.*) showed a C_max_ of 4.01 μg/mL (see further sections), which was below the calculated safety margin. Thus, we concluded that the compound CPL304110 did not pose a risk in terms of cardiac safety. Of note, although an overall false-negative rate of the hERG Predictor™ kit is low (as stated by the manufacturer), this assay can be considered a screening approach, and a follow-up patch clamp experiment must be performed in case of further development of the inhibitor. Furthermore, due to the nature of the drug development process, the presented calculations combine human and rodent characteristics and need to be further investigated as soon as human pharmacokinetic data become available.

### CPL304110 shows a very good safety profile

CPL304110 was active in the Diversity PLUS panel with 64 annotated readouts across the 12 systems. CPL304110 had detectable cytotoxicity in the MyoF system at the top concentration (10 μM) and was antiproliferative to human primary B cells (10 μM and 3.3 μM), coronary artery smooth muscle cells (10 μM, 3.3 μM, and 1.1 μM), endothelial cells (10 μM, 3.3 μM, 1.1 μM, and 370 nM), and fibroblasts (10 μM, 3.3 μM, and 1.1 μM). Broad antiproliferative effects on multiple cell types, especially endothelial cells, are commonly documented for compounds developed for oncology, but not for autoimmune indications. However, CPL304110 was also highly active in systems modeling immune cell activation, including the lipopolysaccharide (LPS) system (monocyte activation), SAg system (T-cell activation), BT system (B-cell activation), and the Mphg system (macrophage activation). CPL304110 inhibited B-cell proliferation and activation (BT system) but did not impact T-cell proliferation (SAg system). CPL304110 was also highly active in systems modeling different types of vascular inflammation, including the 3C system (Th1 type), 4H system (Th2 type), and CASM3C, and in the system modeling coronary artery smooth muscle inflammation. CPL304110 inhibited tissue remodeling factors in the HDF3CGF (system modeling wound healing) as well as in the MyoF (system modeling fibrosis-related biology). CPL304110 was the least active in systems containing epithelial cells (BF4T and BE3C) and keratinocytes (KF3CT).

Overlay of CPL304110 FGFR inhibitor with AZD4547 (selective FGFR inhibitor targeting FGFR1/2/3) identified 32 common activities, eight of which were detected in the system HDF3CGF that models wound healing. Profiles shared a similar pattern overall, with the SAg and BT systems demonstrating the most differences. Of note, both CPL304110 and AZD4547 were cytotoxic in the MyoF system at 10 μM. Similarity analysis identified the PKA inhibitor H-89 as the most similar profile, with Pearson’s correlation coefficient (*r* = 0.839) above our determined threshold (*r* ≥ 0.7), indicating that these compounds share a mechanistically relevant similarity. At the highest non-cytotoxic concentration, the most similar profile was the c-Met inhibitor, MK-2461. However, Pearson’s correlation coefficient (*r* = 0.665) was below our determined threshold (*r* ≥ 0.7).

SAFETYscan47 Panel screening platform was used to measure interactions between 10 µM CPL304110 and 78 targets. A broad *in vitro* SAFETYscan47 Panel screening, composed of different assays including GPCR cAMP Modulation, Calcium Mobilization, Nuclear Hormone Receptor, KINOMEscan Binding, Ion Channel, and Transporter and Enzymatic assay, was conducted to detect major potential adverse activity of CPL304110 that would predict clinical effects. SAFETYscan47 Panel consists of over 78 tests, ranging from molecular assays to cell-based models, through proof-of-concept *in vivo* activity determinations. As a result, we found that CPL304110 at the concentration of 10 μM affects the activity of ion channels. CPL304110 compound inhibited the activity of nAChR(a4/2b) (81.6%) and HTR3A (82.0% of response). Also, the three kinases from the kinase groups demonstrated ≥98% inhibition of their kinase activity: LCK (101.7%), VEGFR2 (99.8%), and INSR (98.0% of response). The secondary screening of the CPL304110 compound at the concentration of 10 μM revealed that one of the non-kinase enzymes, COX1, demonstrated ≥80% inhibition of enzyme activity (**Supplementary Table 4**). CPL304110 at the concentration of 10,000 nM presented agonist activity against CNR2 gene from the GPCR group on the level of 80% response, which may suggest an inverse agonist mechanism of action (**Supplementary Table 4**).

### *In vivo* experiments showed a favorable PK/PD profile of CPL304110

**Figure 7:**
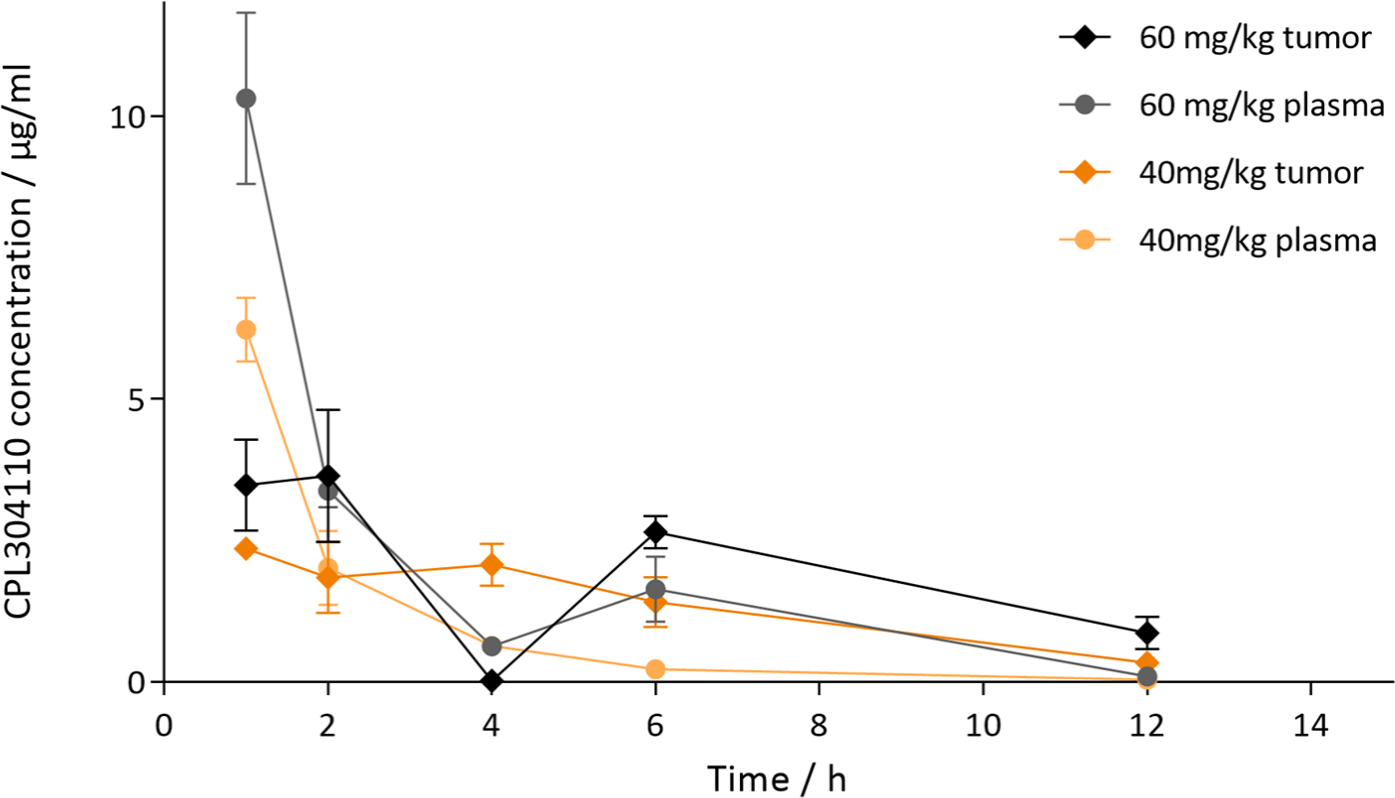
PK/PD analysis of single oral administration CPL304110 or vehicle in mice bearing RT-112 tumors. The compound was administered in two doses (40 or 60 mg/kg), and plasma and tumors were collected 1, 2, 4, 6, and 12 h after the treatment.

A single dose of CPL304110 was administered orally to mice bearing RT-112 tumors to assess PK/PD. In this study, a decrease in the concentration of CPL304110 in the tumor was observed over time, but a dose of 40 mg/kg CPL304110 was maintained up to 12 h in concentrations exceeding its IC_50_ values obtained for the RT-112 line (0.048 µg/mL, i.e., 106 nM) (**Figure 7**).

### CPL304110 shows antitumor activity in different human cell line xenograft models

**Figure 8:**
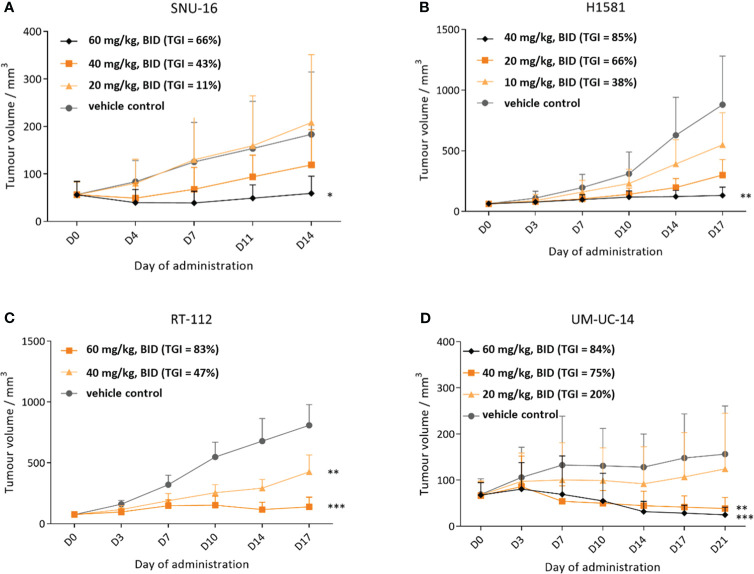
Efficacy of CPL304110 in different cancer cell-line xenograft models with FGFR alterations. **(A)** SNU-16 (FGFR2-amplified) gastric cancer model. **(B)** H1581 (FGFR1-amplified) lung cancer model. **(C)** RT-112 (FGFR3-fused and amplified) bladder cancer model. **(D)** UM-UC-14 (FGFR3-mutated) bladder cancer model. Data are presented as means + SEM for each group of mice (n = 12 for **(A, C, D)** models and n = 6 mice for **(B)** model); ANOVA with *post-hoc* Dunnett’s test *vs.* vehicle-treated, *p < 0.05; **p < 0.01; ***p < 0.001. SEM, standard error of the mean; BID, twice a day.

The antitumor activity of CPL304110 was investigated in the following four subcutaneous xenograft tumor models with confirmed genetic alterations in FGFR genes: FGFR1-overexpressing lung xenografts (H1581), FGFR2-amplified gastric xenograft (SNU16), and two FGFR3-dependent bladder xenografts models (RT-112 and UM-UC-14) (**Figure 8**). Compound and vehicle were administered orally twice daily (BID) at doses in the range of 10–60 mg/kg. As shown in **Figure 8A**, CPL304110 inhibited tumor growth of the SNU-16 model with amplification of FGFR2 in the two highest treatment groups (40 and 60 mg/kg) in a dose-dependent manner. The TGI effect observed for the dose of 60 mg/kg in comparison to the vehicle control group was statistically significant. At termination (day 14), the TGI value for the highest dose was 66%. A more potent effect of CPL304110 was found in the H1581 model with confirmed FGFR1 amplification. A significant decrease in tumor size was observed in the 40 mg/kg group of CPL304110 starting from day 14 of the study (**Figure 8B**) until the last day of treatment (day 17), with a TGI of 85%. Next, the activity of CPL304110 was evaluated against an FGFR3-dependent model—RT-112 bladder carcinoma carrying the FGFR3-TACC3 fusion. Significant inhibition of tumor growth was observed starting from day 3 and day 7 at doses of 60 and 40 mg/kg BID, respectively (**Figure 8D**), with TGI values on day 17 calculated as 83% and 47%, respectively. Finally, the activity of CPL304110 in the second tumor model was also tested using a mutation in the FGFR3 gene—bladder cancer xenografts UM-UC-14. This xenograft model showed a significant tumor growth inhibition at doses of 40 and 60 mg/kg starting from day 10 to the end of the study, with TGI of 75% (dose 40 mg/kg, BID) and 84% (dose 60 mg/kg, BID) on day 21 (**Figure 8D**). Moreover, no significant change in body weight, no general toxicity (considered as morbidity or unspecific clinical signs), and no change in hematological parameters were observed in any of the four human cell-line xenograft models (data not shown). Taken together, the results of *in vivo* efficacy studies on cell-line xenograft tumor models with different FGFR aberrations show that CPL304110 exhibits a potent broad spectrum of antitumor activity against FGFR1, 2, and 3, which represent different types of tumor histology in the clinical setting.

### CPL304110 suppresses the growth of patient-derived tumor xenograft

**Figure 9:**
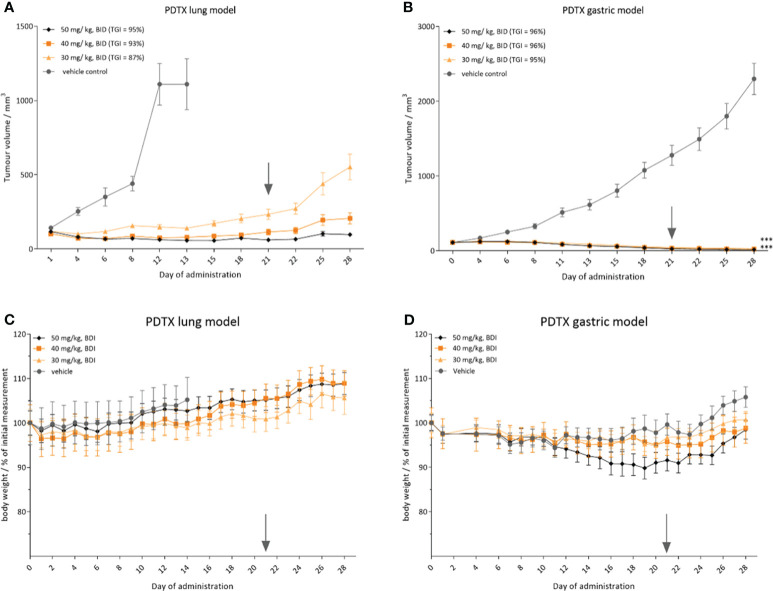
CPL304110 presents antitumor activity in patient-derived tumor models with FGFR2 amplification. Tumor volume in **(A)** small cell lung cancer PDTX model (LU6429) and **(B)** gastric cancer PDTX model (GA1224); relative body weight in **(C)** LU6429 model and **(D)** GA1224 model. Data are presented as means + SEM for each group of mice (n = 10); ANOVA with *post-hoc* Dunnett’s test *vs.* vehicle-treated, ***p < 0.001. SEM, standard error of the mean; BID, twice a day. The gray arrow marks the last day of the treatment.

To assess the potential clinical utility of CPL304110, *in vivo* efficacy in two patient-derived xenograft models was evaluated with confirmed FGFR2 amplification, i.e., in a non-small cell lung cancer PDTX model LU6429 and a gastric PDTX model GA1224. When the mean tumor volume reached volume >100 mm^3^, the mice were randomized into treatment groups using a stratified randomization method. Treatment was scheduled for 3 weeks, twice daily, followed by the 7-day recovery period for each PDTX model. During the treatment phase, there was a statistically significant tumor growth inhibition in groups treated with CPL304110 orally, twice a day at 30, 40, and 50 mg/kg (two-way ANOVA, p < 0.001) in the lung LU6429 model (**Figure 9A**). During the 7-day recovery period, tumor regrowth was observed in all treatment groups in a dose-dependent manner. An even more noticeable effect of CPL304110 was observed in the gastric PDTX model GA1224, where the compound exhibited stronger tumor-suppressive activity at all doses. During the treatment phase, tumor regression was observed in all treatment groups at all three doses (30, 40, and 50 mg/kg, BID) compared with the vehicle group (two-way ANOVA, p < 0.001) (**Figures 9A, B**). During the 7-day treatment-free observation period, no tumor regrowth was observed in any of the treatment groups in the case of the GA1224 model. Importantly, CPL304110 at all three dose levels was generally well-tolerated in both MF-1 (**Figure 9C**, LU6429 model) and BALB/c nude (**Figure 9D**, GA1224 model) mice. No significant loss in body weight (**Figures 9C, D**) or statistically significant adverse clinical signs were observed during the dosing phase; only one mouse from the lowest dose group was found dead on day 21 (the day of experiment termination). Necropsy did not show any abnormalities. Since the GA1224 model is a cachexic model, moderate BLW was observed in the vehicle group as well as in the treatment groups (**Figure 9D**). Taken together, oral administration of CPL304110 resulted in strong inhibition of growth of both patient-derived FGFR2-dependent tumor xenografts.

## Discussion

There is increasing evidence that aberrant FGFR signaling plays a key role in tumorigenesis and cancer progression. Currently developed small chemical inhibitors targeting the FGFR pathways offer a novel and effective strategy for the therapeutic intervention in cancers driven by FGFR aberrations. Lately, there has been progress in the development of selective FGFR inhibitors (31), leading to less toxic compounds with fewer side effects (4). Some of the currently developed inhibitors show promising efficacy and are being evaluated in clinical trials. Recently, erdafitinib and pemigatinib, pan-FGFR inhibitors, have been approved by the Food and Drug Administration for the treatment of metastatic urothelial carcinoma and cholangiocarcinoma, respectively (4). However, there are still challenges in the field of FGFR inhibitor development, including the need for more selective and potent biomarker-driven FGFR inhibitors.

In this study, we described for the first time *in vitro* and *in vivo* data for the new highly potent and selective FGFR1–3 kinase inhibitor CPL304110, which is based on the novel chemical scaffold (16). The potency of FGFR inhibition was confirmed in biochemical kinase domain binding and activity assays. We demonstrated that CPL304110 binds to FGFR1–3 receptors with low nanomolar affinity and strongly inhibits FGFR1–3 kinase activity with IC_50_ values in the nanomolar range. The SPR results suggest different binding profiles of compound CPL304110 in the catalytic pocket of individual FGFR kinases, with very long residence times for FGFR1 and FGFR2 kinase domains. Presented binding profiles are in line with computer models of complex formation, in which differences in the topology of kinase domains of FGFR2 and FGFR1/3 lead to a better exposition of E571 residue, and a formation of a salt bridge between CPL304110 and FGFR1/3, leading to much more stable complex. This interaction has a strong covalent contribution, which can mimic covalently stabilized inhibitors, and it may explain the formation of more stable complexes of CPL304110 with FGFR1 and FGFR3, than with FGFR2, which may result in different inhibition patterns of different FGFRs. KINOMEscan™ profiling of 100 and 468 kinase targets showed favorable selectivity scores for both binding and inhibition. CPL304110 at the higher concentration on the panel of 468 kinases inhibited, in addition to the FGFR kinases, the activity of 38 other kinases or disease-relevant kinase mutants with ≥90% efficiency. These kinases include RET, FLT3, KIT, and members of the VEGFR, PDGFR, and JAK families. Particularly, the CPL304110 compound had the strongest inhibitory activity against CSF1R, FLT3 (D835V), KIT (A829P), RET, RIPK1, TRKA, TYK2, KIT (V559D), and RET (M918T). Moreover, CPL304110 inhibited the FGFR3 variant with the activating G697C mutation, which was observed in 62% of the examined OSCC. It is possible that the activating potential of this mutation could be context-dependent and may involve engaging other genes (32). In summary, our compound also showed an inhibitory activity toward some particular kinases, which either have a slight influence on the safety or have a beneficial impact, suggesting that the tested compound could be a promising agent for the treatment of patients with cancers with specified mutations. Moreover, CPL304110 presented strong selective antiproliferative activity against cancer cell lines with aberrations of FGFR. The subsequent results showed a strong dependence of the response to CPL304110 in tumor cell lines on the presence of genetic aberrations in the FGFR. Likewise, the data from oral administration of CPL304110 confirmed strong antitumor efficacy in FGFR-dependent xenograft models. This is in agreement with reports showing that FGFR changes are the determinant of sensitivity to FGFR inhibition, and the response depends on the type of aberrations (33). For example, data presented by Grünewald et al. (34) showed that not all cell lines overexpressing one of the FGFR subtypes are sensitive to FGFR inhibition, which can suggest that other oncogenes or oncogenic pathways, in addition to FGFR signaling, may be dominant oncogenic drivers in these cells. Interestingly, in our study both, the CPL304110-sensitive cell lines and the *in vivo* models in which inhibition of tumor growth was observed had one or more FGFR aberrations. Thus, the observed effects on cell proliferation and tumor growth strongly suggest that CPL304110 inhibits FGFR activation-induced cancer growth in a dose-dependent manner with excellent efficacy in cancers highly expressing FGFR. These observations further confirm the need to use the presence of FGFR aberrations in the tumor as a stratification biomarker for patients in clinical trials.

We found certain advantages of CPL304110 over two already approved treatment FGFR inhibitors: erdafitinib and pemigatinib.

Structurally, erdafitinib, classified as a quinoxaline derivative, and pemigatinib, identified as a tetra-azatricyclotridecatetraene derivative, represent distinct chemical classes. This distinction is further highlighted when compared with CPL304110 and AZD4547, both of which fall under the category of pyrazole derivatives. Given these chemical differences, we selected AZD4547 as the reference compound for our analyses, rather than erdafitinib or pemigatinib. These structural variations lead to notable differences in how these compounds interact with FGFR kinases (**Supplementary Figure 1**). Erdafitinib and PEMIGATINIB, for instance, are limited in their interaction with the A564 amino acid within the hinge region of FGFR1 (based on comparison of crystal structures—PDB ID: 5EW8 and 7WCL, respectively). In contrast, the interaction pattern of CPL304110 and AZD4547 (PDB ID: 4RWJ) is more complex, involving two amino acids, E562 and A564; in addition, CPL304110 interacts with E571. The latter interaction includes the formation of a salt bridge, a strong ionic bond that contributes significantly to the stability of the protein–ligand complex. This enhanced stability was confirmed through SPR analysis. Building on these insights, we found that CPL304110, due to its similar binding profile to AZD4547, could effectively target the FGFR1 (V561M) gatekeeper mutant variant, engaging E531 and M535. This suggests the potential for a broader spectrum of anticancer activity, as detailed in the study by Roskoski et al. (35).

Functionally, the FDA has approved FGFR inhibitors such as erdafitinib and pemigatinib as innovative treatment regimens for patients with bladder cancer and cholangiocarcinoma, respectively, with appropriate FGFR molecular alterations. Erdafitinib received accelerated approval, based on clinical Phase II results, for the treatment of patients with advanced or metastatic urothelial carcinoma with susceptible FGFR3 or FGFR2 genetic alterations (36). Pemigatinib received accelerated approval for treating patients with locally advanced or metastatic cholangiocarcinoma harboring an FGFR2 fusion or other rearrangements. This approval was based on efficacy and safety results from a Phase II registration trial (37). However, when comparing the Phase I results of both of these approved drugs with preliminary Phase IA data from the CPL304110 inhibitor, the promising therapeutic potential of CPL304110 and its outstanding safety profile can be observed. The main aim of these trials was to establish the safety of the candidates for anticancer drugs. In the case of erdafitinib, the most common treatment-emergent adverse events (TEAEs) were hyperphosphatemia (65%), asthenia (55%), dry mouth (45%), nail toxicity (35%), constipation (34%), decreased appetite (32%), and dysgeusia (31%), and simultaneously, 42% of patients experienced grade ≥ 3 TEAE (38). A similar trend was observed in the Phase I trial of pemigatinib, where hyperphosphatemia was the most frequently reported TEAE (75.0%), with grade ≥ 3 occurring in 2.3% of patients. Additionally, grade ≥ 3 fatigue occurred in 10.2% of patients (39). Under the examination of the Phase IA safety results of CPL304110, the most common TEAEs were anemia (19%), ocular toxicity (19%), and dry eye (14.3%), and all AEs mentioned were grade 1–2. Only one patient (4.8%) experienced a grade ≥ 3 TEAE (40). Analyzing the clinical efficacy of erdafitinib, it can be noted that out of FGFR-dependent patients (N=23), only four of them achieved partial response (PR), which constitutes only 17.4% of such therapeutic responses (38), and in the case of pemigatinib, the PR rate was 9.4% (39). At the same time, the therapeutic response of the CPL304110 inhibitor in the Phase IA trial reached the threshold level of PR rate of 50% (authors’ correction due to an editorial error in the numerical value) among patients with a confirmed FGFR molecular change (40). This undoubtedly represents a significant and promising result of the effectiveness of the CPL304110 inhibitor, along with its overall good tolerability.

The activity and selectivity of CPL304110 in preclinical analysis qualify it for consideration as a drug with a wide therapeutic window and limited side effects in clinical use for patients with FGFR aberrations. CPL304110 is currently under clinical investigation (Phase I: NCT4149691, 01FGFR2018); after completing the part without molecular selection of patients, the following part will include the selection of patients with chosen aberrations in *FGFR1*, *2*, and *3* genes in lung, stomach, and bladder cancers, respectively. By testing the compound in these different FGFR-relevant tumor models, we also hope to identify the specific types of tumor histology that can be effectively targeted by CPL304110 for further patient selection in clinical studies.

## Data availability statement

The datasets presented in this study can be found in online repositories. The names of the repository/repositories and accession number(s) can be found in the article/**Supplementary Material**.

## Ethics statement

Ethical approval was not required for the studies on humans in accordance with the local legislation and institutional requirements because only commercially available established cell lines were used. The animal study was approved by Bioethics Committee of the Hirszfeld Institute of Immunology and Experimental Therapy. The study was conducted in accordance with the local legislation and institutional requirements.

## Author contributions

DP: Conceptualization, Supervision, Writing – original draft, Data curation, Writing – review & editing, Formal analysis, Investigation. AS: Conceptualization, Investigation, Data curation, Supervision, Writing – review & editing, Formal analysis, Methodology, Funding acquisition, Project administration. MS: Investigation, Writing – review & editing, Validation. AM: Investigation, Writing – review & editing, Data curation, Formal analysis. PS: Investigation, Writing – review & editing, Conceptualization, Validation, Formal analysis, Methodology. FM: Investigation, Writing – original draft, Validation, Writing – review & editing, Methodology. JH: Writing – review & editing, Formal analysis, Investigation. KJ: Investigation, Writing – review & editing, Data curation, Formal analysis, Methodology. DS: Writing – review & editing, Data curation, Formal analysis, Investigation, Methodology, Validation. JD: Writing – review & editing, Data curation, Formal analysis, Investigation, Methodology. MD: Writing – review & editing, Data curation, Formal analysis, Investigation, Methodology. KM: Writing – original draft, Formal analysis. WP: Software, Writing – review & editing, Investigation. DZ: Investigation, Writing – review & editing, Conceptualization. KDz: Investigation, Writing – review & editing. ML: Writing – review & editing, Conceptualization. AY: Conceptualization, Writing – review & editing. PO: Writing – original draft, Resources. NP: Writing – original draft, Resources. KDu: Conceptualization, Writing – review & editing. MW: Conceptualization, Supervision, Writing – review & editing, Funding acquisition, Project administration, Resources. JP: Conceptualization, Writing – original draft, Data curation, Supervision, Writing – review & editing, Project administration.
