# Supplementary material for: Preclinical characterization of CPL304110 as a potent and selective inhibitor of fibroblast growth factor receptors 1, 2, and 3 for gastric, bladder, and squamous cell lung cancer
Source: Front Oncol. 2024 Jan 12;13:1293728. doi: 10.3389/fonc.2023.1293728 (PMC10811212; doi:10.3389/fonc.2023.1293728)
Supplement: Supplementary file 1 [file DataSheet_1.zip › Supplement Table 1 Metabolic Stability.docx]

**Supplement Table 1.** **MS detection parameters for LC-MS metabolic stability assessment**

|  | **verapamil** | **warfarin** | **CPL304110** |
| --- | --- | --- | --- |
| **monoisotopic mass** | 454.28 | 308.10 | 446.26 |
| **MRM transitions** | 455.4 🡪 465.2 | 309.1 🡪 251.1 | 447.2 🡪 296.2 |
| **declustering potential** | 150 | 135 | 200 |
| **collision energy** | 36 | 29 | 54 |
| **collision cell exit potential** | 10 | 10 | 18 |
