# Supplementary material for: Preclinical characterization of CPL304110 as a potent and selective inhibitor of fibroblast growth factor receptors 1, 2, and 3 for gastric, bladder, and squamous cell lung cancer
Source: Front Oncol. 2024 Jan 12;13:1293728. doi: 10.3389/fonc.2023.1293728 (PMC10811212; doi:10.3389/fonc.2023.1293728)
Supplement: Supplementary file 1 [file DataSheet_1.zip › Supplement Table 2 List of the Cell Lines.docx]

**Supplement Table 2. List of cell lines**

| **Cell line** | **Cat no. / manufacturer** | **Tissue** | **FGFR alteration** |
| --- | --- | --- | --- |
| **FGFR-dependent tumor cell lines** | | | |
| NCI-H1581 |  | lung | FGFR1 amplification  FGFR3 mutation N549K |
| SNU-16 |  | gastric | FGFR2 amplification |
| RT-112/84 |  | bladder | FGFR3:TACC3 fusion  FGFR3 amplification |
| KATO III | HTB-103, ATCC | gastric | FGFR2 amplification |
| UM-UC-14 |  | bladder | FGFR3 mutation S249C |
| AN3CA | HTB-111, ATCC | uterus | FGFR2 amplification |
| KMS 11 | JCRB1179, JRCB | myeloma | IgH-FGFR3 translocations |
| SW-780 | CRL-2169, ATCC | bladder | FGFR3:BAIAP2L1 fusion |
| NCI-H520 | HTB-182, ATCC | lung | FGFR1 amplification |
| CAL120 | ACC 459, DSMZ | breast | FGFR1 amplification |
| **FGFR-independent tumor cell lines** | | | |
| WSU-NHL | ACC 58, DSMZ | lymphoma | - |
| HEL 92.1.7 | TIB-180, ATCC | bone marrow | - |
| HepG2 | HB-8065, ATCC | liver | - |
| HCT116 | CCL-247, ATCC | colon | - |
| HeLa | CCL-2, ATCC | uterus | - |
| U937 | ACC 5, DSMZ | lymphoma | - |
| T24 | ACC 376, DSMZ | bladder | - |
| LoVo | ACC 350, DSMZ | colon | - |
| H460 | HTB-177, ATCC | lung | - |
| PC-3 | ACC 465, DSMZ | prostate | - |
| **Normal cell lines** | | | |
| HUVEC | PCS-100-010, ATCC | umbilical vein | - |
