# Supplementary material for: Preclinical characterization of CPL304110 as a potent and selective inhibitor of fibroblast growth factor receptors 1, 2, and 3 for gastric, bladder, and squamous cell lung cancer
Source: Front Oncol. 2024 Jan 12;13:1293728. doi: 10.3389/fonc.2023.1293728 (PMC10811212; doi:10.3389/fonc.2023.1293728)
Supplement: Supplementary file 1 [file DataSheet_1.zip › Supplement Table 3 BioMAP Profiling.docx]

Supplement Table 3. List of 12 systems in the BioMAP Diversity PLUS panel, with cell types, disease context and list of biomarker readouts optimized for system.

| System name | Human cell types | Stimulation | Disease/tissue relevance |
| --- | --- | --- | --- |
| 3C | Venular endothelial cells | TNFα, IL-1β, IFNγ | Cardiovascular disease, chronic inflammation |
| 4H | Venular endothelial cells | IL-4, histamine | Autoimmnity, allergy, asthma |
| LPS | Venular endothelial cells, PBMC | TLR4 ligand | Chronic inflammation, cardiovascular disease |
| SAg | Venular endothelial cells, PBMC | TCR ligands (1X) | Chronic inflammation, autoimmune disease |
| BT | PBMC, B cells | Α-IgM, TCR ligands (0.001X, sub-mitogenic levels) | Asthma, oncology, autoimmunity, allergy |
| BF4T | Bronchial epithelial cells, dermal fibroblasts | IL-4, TNFα | Fibrosis, lung inflammation, asthma, allergy |
| BE3C | Bronchial epithelial cells | IL-1β, IFNγ, TNFα | COPD, lung inflammation |
| CASM3C | Coronary artery smooth muscle cells | IL-1β, IFNγ, TNFα | Cardiovascular inflammation, restenosis |
| HDF3CGF | Dermal fibroblasts | IFNγ, TNFα, IL‑1β, EGF, bFGF, PDGF‑BB | Fibrosis chronic inflammation |
| KF3CT | Keratinocytes, dermal fibroblasts | IL-1β, IFNγ, TGFβ, TNFα | Dermatitis, psoriasis |
| MyoF | Lung fibroblasts | TGFβ, TNFα | Wound healing, matrix remodeling, fibrosis, chronic inflammation |
| /Mphg | Macrophages, venular endothelial cells | TLR2 ligand | Chronic inflammation, restenosis, cardiovascular disease |
