# Supplementary material for: Preclinical characterization of CPL304110 as a potent and selective inhibitor of fibroblast growth factor receptors 1, 2, and 3 for gastric, bladder, and squamous cell lung cancer
Source: Front Oncol. 2024 Jan 12;13:1293728. doi: 10.3389/fonc.2023.1293728 (PMC10811212; doi:10.3389/fonc.2023.1293728)
Supplement: Supplementary file 1 [file DataSheet_1.zip › Supplement Table 4 CPL304110 SaftyScan-47 Panel Data.docx]

Supplement Table 4. CPL304110 dates from SaftyScan-47 panel.

| **Gene Symbol** | **Assay Mode** | **CPL304110 10 µM % Response** |
| --- | --- | --- |
|  |  |  |
| **GPCRs** | | **Average** |
| ADORA2A | Agonist | -2,9 |
| ADORA2A | Antagonist | 6,1 |
| ADRA1A | Agonist | -1,4 |
| ADRA1A | Antagonist | 7,1 |
| ADRA2A | Agonist | -20,1 |
| ADRA2A | Antagonist | 30,7 |
| ADRB1 | Agonist | -0,3 |
| ADRB1 | Antagonist | -33,8 |
| ADRB2 | Agonist | -1,1 |
| ADRB2 | Antagonist | 3,5 |
| AVPR1A | Agonist | 1,6 |
| AVPR1A | Antagonist | -22,5 |
| CCKAR | Agonist | -1,8 |
| CCKAR | Antagonist | -6,9 |
| CHRM1 | Agonist | -0,1 |
| CHRM1 | Antagonist | 47,7 |
| CHRM2 | Agonist | -13,3 |
| CHRM2 | Antagonist | 25,9 |
| CHRM3 | Agonist | -4,0 |
| CHRM3 | Antagonist | 19,6 |
| CNR1 | Agonist | -8,5 |
| CNR1 | Antagonist | 2,3 |
| CNR2 | Agonist | -80,3 |
| CNR2 | Antagonist | -3,0 |
| DRD1 | Agonist | 1,5 |
| DRD1 | Antagonist | 31,0 |
| DRD2S | Agonist | 0,6 |
| DRD2S | Antagonist | 0,1 |
| EDNRA | Agonist | -1,4 |
| EDNRA | Antagonist | 11,4 |
| HRH1 | Agonist | 0,8 |
| HRH1 | Antagonist | -0,9 |
| HRH2 | Agonist | -0,3 |
| HRH2 | Antagonist | -18,8 |
| HTR1A | Agonist | 1,2 |
| HTR1A | Antagonist | 14,1 |
| HTR1B | Agonist | -6,1 |
| HTR1B | Antagonist | 48,5 |
| HTR2A | Agonist | -2,1 |
| HTR2A | Antagonist | 38,6 |
| HTR2B | Agonist | -8,1 |
| HTR2B | Antagonist | 31,5 |
| OPRD1 | Agonist | -4,7 |
| OPRD1 | Antagonist | 7,1 |
| OPRK1 | Agonist | 17,3 |
| OPRK1 | Antagonist | -2,3 |
| OPRM1 | Agonist | -16,8 |
| OPRM1 | Antagonist | 10,6 |
| **Nuclear Hormone Receptors** | | **Average** |
| AR | Agonist | -0,6 |
| AR | Antagonist | 6,6 |
| GR | Agonist | -0,3 |
| GR | Antagonist | 24,5 |
| **Transporters** | | **Average** |
| DAT | Blocker | 65,3 |
| NET | Blocker | 31,3 |
| SERT | Blocker | 26,9 |
| **Ion Channels** | | **Average** |
| CAV1.2 | Blocker | -11,1 |
| GABAA | Opener | -12,9 |
| GABAA | Blocker | 6,7 |
| hERG | Blocker | 5,8 |
| HTR3A | Opener | -0,6 |
| HTR3A | Blocker | 82,0 |
| KvLQT1/minK | Opener | -6,1 |
| KvLQT1/minK | Blocker | 2,6 |
| nAChR(a4/b2) | Opener | 1,6 |
| nAChR(a4/b2) | Blocker | 81,6 |
| NAV1.5 | Blocker | 8,2 |
| NMDAR (1A/2B) | Opener | -6,1 |
| NMDAR (1A/2B) | Blocker | 16,9 |
| **Non-Kinase Enzymes** | | **Average** |
| AChE | Inhibitor | 25,9 |
| COX1 | Inhibitor | 79,8 |
| COX2 | Inhibitor | 62,9 |
| MAOA | Inhibitor | 0,4 |
| PDE3A | Inhibitor | 0,5 |
| PDE4D2 | Inhibitor | -5,0 |
| **Kinases** | | **Average** |
| INSR | Inhibitor | 98,0 |
| LCK | Inhibitor | 101,7 |
| ROCK1 | Inhibitor | 21,0 |
| VEGFR2 | Inhibitor | 99,8 |
