# Supplementary material for: Preclinical characterization of CPL304110 as a potent and selective inhibitor of fibroblast growth factor receptors 1, 2, and 3 for gastric, bladder, and squamous cell lung cancer
Source: Front Oncol. 2024 Jan 12;13:1293728. doi: 10.3389/fonc.2023.1293728 (PMC10811212; doi:10.3389/fonc.2023.1293728)
Supplement: Supplementary file 1 [file DataSheet_1.zip › Supplement Table 5 PhysChem Properties.docx]

**Supplement Table 5A. Physicochemical characterization of CPL304110.**

I. Physicochemical properties of CPL304110

| pH | Distribution coefficient logD | Thermodynamic solubility [mg/mL] |
| --- | --- | --- |
| 1.2 | 0.44 | 39.30 |
| 4.2 | 1.79 | 3.10 |
| 6.8 | 3.49 | 0.02 |
| 7.4 | 4.22 | 0.01 |
| 12.8 | 3.49 | 0.06 |
|  |  |  |

II. Permeability of CPL30411001; data are mean from independent replicates, ± SD

| **compound** | **Perm*10^6^ [cm/s]** |
| --- | --- |
| **atenolol** | 0.010 ± 0.008 |
| **testosterone** | 32.3 ± 13.7 |
| **CPL304110** | 0.813 ± 0.248 |

**Description of methodology of assays**

**Thermodynamic solubility**

Thermodynamic solubility was determined by shake-flask protocol [1], [2], [3]. Incubation appropriate amount of solid compound was performed in appropriate aqueous buffers (pH 1.2 – 0.1 M HCl aq, pH 4.5 – 10 mM acetate buffer, pH 6.8 – 0.1 M phosphate buffer, pH 7.4 - 0.1 M phosphate-buffered saline, pH 12.8 – 0.1 M NaOH aq) at 25° C with stirring at 500 rpm. The samples were taken at the start time and after 24h of incubation, filtered through 0.22 µm PTFE syringe filters and diluted with 2 volumes of acetonitrile. Sample concentrations were determined by UHPLC-UV/Vis. A calibration curve was prepared to quantify the contents of the compound in the test solution.

Details of the chromatography conditions are shown in the tables below (Supplement Table 5B).

| **Supplement Table 5B. Parameters of HPLC analysis for kinetic solubility test** | | | | |
| --- | --- | --- | --- | --- |
| **Instrument** | High-performance liquid chromatography 1290 Infinity II,  Agilent Technologies | | | |
| **Chromatographic column** | Waters Acquity BEH 2.1 x 50 mm; 1.7 µm | | | |
| **Thermostat temperature** | 40°C | | | |
| **Autosampler temperature** | 10°C | | | |
| **Mobile phases** | WATER | A: 10 mM HCOONH_4_ pH 3.2 in H_2_O | | |
|  | ORGANIC | B: ACN | | |
| **Method** | Time [min] | Flow [mL/min] | %A | % B |
|  | Initial | 0.60 | 100 | 0.00 |
|  | 0.30 | 0.60 | 100 | 0.00 |
|  | 0.60 | 0.60 | 80.0 | 20.0 |
|  | 1.20 | 0.60 | 1.00 | 99.0 |
|  | 1.90 | 0.60 | 1.00 | 99.0 |
|  | 1.95 | 0.60 | 100 | 0.00 |
|  | 2.50 | 0.60 | 100 | 0.00 |
| **Injection volume** | 3 µL | | | |

**Distribution coefficient (logD)**

Determination of distribution coefficient (logD) based on shake-flask methodology [4-5]. CPL304110 has been incubated at a concentration of 500 µM in the 1-octanol:buffer 50:50 (v/v) solution over 24 h at 25° C with stirring at 500 rpm. Appropriate buffer solutions were used: pH 1.2 – 0.1 M HCl aq, pH 4.5 – 10 mM acetate buffer, pH 6.8 – 0.1 M phosphate buffer, pH 7.4 - 0.1 M phosphate-buffered saline, pH 12.8 – 0.1 M NaOH aq. The analyte concentrations in study samples were determined a base of calibration curves by the HPLC-UV method (see table 2) or LC-MS/MS method (see table 3.) logD is calculated by the following equation:

$$\log D= \log(\frac{c_{1-octanol}}{c_{buffer}})$$

where:

$c_{1-octanol}$ is compound concentration in 1-octanol fraction

$c_{buffer}$ is compound concentration in buffer fraction

| **Supplement Table 5C. Parameters of LC-MS/MS method for determination of distribution coefficient** | | | | |
| --- | --- | --- | --- | --- |
| **Instrument** | Waters Acquity TQD detector combined with H-Class UHPLC liquid chromatography system | | | |
| **Chromatographic column** | Waters Acquity BEH 2.1 x 50 mm; 1.7 µm | | | |
| **Thermostat temperature** | 40°C | | | |
| **Autosampler temperature** | 10°C | | | |
| **Mobile phases** | WATER | A: 10 mM HCOONH_4_ pH 3.2 in H_2_O | | |
|  | ORGANIC | B: ACN | | |
| **Method** | Time [min] | Flow [mL/min] | %A | % B |
|  | Initial | 0.60 | 100 | 0.00 |
|  | 0.30 | 0.60 | 100 | 0.00 |
|  | 0.60 | 0.60 | 80.0 | 20.0 |
|  | 1.20 | 0.60 | 1.00 | 99.0 |
|  | 1.90 | 0.60 | 1.00 | 99.0 |
|  | 1.95 | 0.60 | 100 | 0.00 |
|  | 2.50 | 0.60 | 100 | 0.00 |
| **Injection volume** | 3 µL | | | |
| **Mass spectrometer** |  | | | |

**Parallel Artificial Membrane Permeability Assay (PAMPA)**

An artificial membrane was prepared in 96-well filter plates (Millipore MultiScreen IP Filter Plate). Each well of the donor plate was coated with 5 µL of the 2% lecithin/dodecane solution. Next, in each well of the donor plate, 150 µL of a compound-containing donor solution (compound dissolved in 5% DMSO, PBS) was added. All the compounds were added at a concentration of 10 µM. The compounds were prepared from 10 mM stock solutions in DMSO. The 300 µL of buffer (5% DMSO in PBS, pH 7.4) was transferred to each well of the PTFE acceptor plate in technical triplicates. The compound-filled donor plate was placed into the acceptor plate. Incubation was carried out for 4 h at room temperature.

Samples were taken after incubation from donor and acceptor compartments, diluted with 2 volumes of acetonitrile containing a 200 nM concentration of the internal standard (imipramine). The solutions from the donor plate were diluted if necessary. Testosterone (10 µM) and atenolol (10 µM) were used as high and low reference permeability compounds, respectively.

The compound concentration was determined by LC-MS/MS (an Agilent 6460 MS/MS mass spectrometer equipped with Agilent Infinity II 1290 UHPLC, Agilent Technologies). A calibration curve was prepared to quantify the contents of the compound in the test solution [4, 5].

1. K. Sugano, A. Okazaki, S. Sugimoto, S. Tavornvipas, A. Omura and T. Mano, “Solubility and Dissolution Profile Assessment in Drug Discovery”, Drug Metab. Pharmacokinet. 22 (4): 225–254, 2007.
2. R. Guhaa, T. S. Dexheimera, A. N. Kestranekb, A. J., A. M. Chervenakb, M. G. Fordb, A. Simeonova, G. P. Rothc, and C. J. Thomasa, “Exploratory Analysis of Kinetic Solubility Measurements of a Small Molecule Library”, Bioorg Med Chem 19(13): 4127–4134, 2011.
3. E. Maes, V. Nikolaev and M. Patek “Kinetic Solubility: Measurement and Data Processing”, Icagen Advancing Early Drug Discovery, 2017.
4. D. Schmidt and J. Lynch, “Evaluation of the reproducibility of Parallel Artificial Membrane Assays (PAMPA)”, Millipore Application Note.
5. J. A. Ruell, A. Avdeef, “Absorption Screening Using the PAMPA Approach”, Methods in Pharmacology and Toxicology Optimization in Drug Discovery: In Vitro Methods, 2004.
