# Supplementary material for: Preclinical characterization of CPL304110 as a potent and selective inhibitor of fibroblast growth factor receptors 1, 2, and 3 for gastric, bladder, and squamous cell lung cancer
Source: Front Oncol. 2024 Jan 12;13:1293728. doi: 10.3389/fonc.2023.1293728 (PMC10811212; doi:10.3389/fonc.2023.1293728)
Supplement: Supplementary file 1 [file DataSheet_1.zip › Supplement Table 6 In vitro Clint & hERG.docx]

| **Supplement Table 6A. *In vitro* *Cl*_int_ of CPL304110; data are mean from independent replicates, ± SD** | | | |
| --- | --- | --- | --- |
|  | ***Cl*_int, HLM_**  / µL·min^-1^·10^-1^ mg protein | ***Cl*_int, MLM_**  / µL·min^-1^·10^-1^ mg protein | ***Cl*_int, MH_**  / µL·min^-1^·10^-6^ cells |
| **verapamil** | 144.9 ± 38.9 | 197.4 ± 13.0 | 77.5 ± 4.9 |
| **warfarin** | 5.7 ± 1.3 | 3.2 ± 1.1 | 2.6 ± 0.19 |
| **CPL304110** | 38.1 ± 13.0 | 50.3 ± 7.0 | 58.5 ± 21.37 |

**Supplement Table 6B. hERG potassium channel binding by CPL30410; data are mean from three independent replicates, ± SD**

|  | **hERG IC_50_** |
| --- | --- |
| **E-4031** | (38 ± 21) nM |
| **CPL304110** | (10.0 ± 8.9) μM |
| **QT interval prolongation safety margin** | 50-fold |
| **CPL304110 safety margin (free drug)** | < 200 nM |
| **CPL304110 fraction unbound, human plasma** | 0.0043 |
| **CPL304110 safety margin (total plasma drug)** | < 46.5 μM  < 20.8 mg/mL |

$$CPL304110 safety margin=\frac{\mathrm{hERG}\mathrm{IC}_{50}}{safety margin\cdot f_{u}}$$

where *f_u_* is fraction unbound in plasma
