# Supplementary figures and images for: Preclinical characterization of CPL304110 as a potent and selective inhibitor of fibroblast growth factor receptors 1, 2, and 3 for gastric, bladder, and squamous cell lung cancer
Source: Front Oncol. 2024 Jan 12;13:1293728. doi: 10.3389/fonc.2023.1293728 (PMC10811212; doi:10.3389/fonc.2023.1293728)

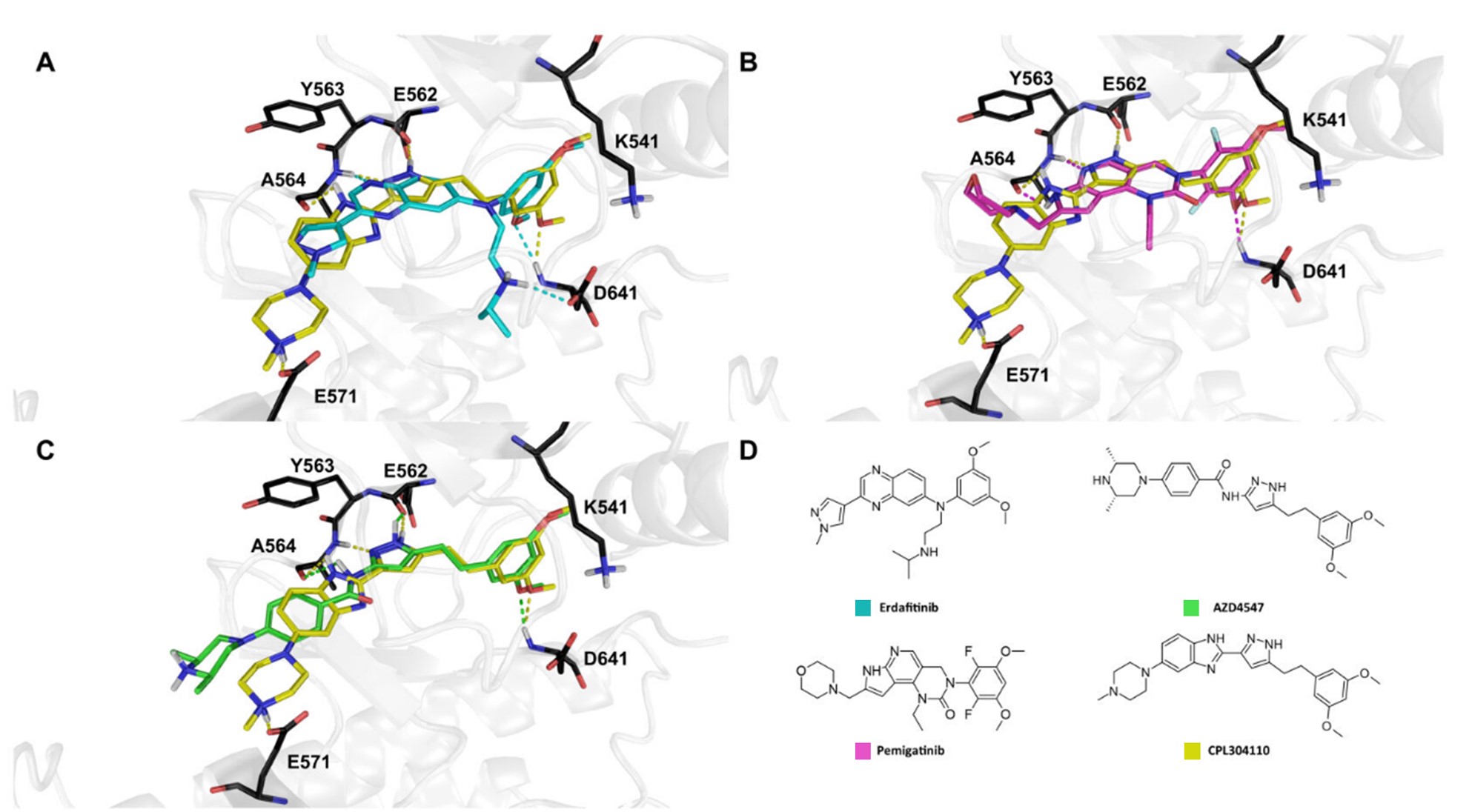

Supplement: Supplementary file 1 [file DataSheet_1.zip › Supplement Figure 1 Molecular Docking.jpg]
